# Evaluating the effect of delayed activation of rapid response teams on patient outcomes: a systematic review protocol

**DOI:** 10.1186/s13643-018-0705-x

**Published:** 2018-03-09

**Authors:** Michael K. Xu, Kathleen G. Dobson, Lehana Thabane, Alison E. Fox-Robichaud

**Affiliations:** 1 Department of Health Research Methods, Evidence, and Impact, McMaster University, DBRI C5-106, 237 Barton St. East, Hamilton, ON L8L 2X2 Canada; 2 Department of Medicine, McMaster University, Hamilton, Canada; 3 Dalla Lana School of Public Health, University of Toronto, Toronto, Canada

**Keywords:** Systematic review, Rapid response team, Critical care, Medical emergency team, Intensive care, Mortality, Delay

## Abstract

**Background:**

Rapid response teams have been widely adopted across the world. Although evidence for their efficacy is not clear, they remain a popular means to detect and react to patient deterioration. This may in part be due to there being no standardized approach to their usage or implementation. A key component of their ability to be effective is the speed of response.

**Objective:**

The objective of this review is to evaluate the effect of delayed response by rapid response teams on hospital mortality (primary), cardiac arrest, and intensive care transfer rates (secondary).

**Methods:**

This review will include randomized and non-randomized studies which examined the effect of delayed response times by rapid response teams on patient mortality, cardiac arrest, and intensive care unit admission rates. This review will include studies of adult patients who have experienced a rapid response team consultation. The search strategy will utilize a combination of keywords and MeSH terms. MEDLINE and Embase will be searched, as well as examining gray literature. Two reviewers will independently screen retrieved citations to determine if they meet inclusion criteria. Studies will be selected that provide information about the impact of response time on patient outcomes.

Comparisons will be made between consults that arrive in a timely manner and consults that are delayed. Quality assessment of randomized studies will be conducted in accordance with guidelines from the Cochrane Handbook for Systematic Reviews of Interventions. Quality assessment of non-randomized studies will be based on the Risk of Bias in Non-randomized Studies-of Interventions (ROBINS-I) assessment tool. Results of the review will be reported according to the Preferred Reporting Items for Systematic Reviews and Meta-Analyses guidelines.

**Discussion:**

This systematic review will identify and synthesize evidence around the impact of delayed response by rapid response teams on patient mortality, cardiac arrest, and intensive care transfer rates.

**Systematic review registration:**

PROSPERO Registration: CRD42017071842.

**Electronic supplementary material:**

The online version of this article (10.1186/s13643-018-0705-x) contains supplementary material, which is available to authorized users.

## Background

Patients exhibit physiological deterioration prior to cardiac arrest [1–4]. Rapid response systems are designed to detect this physiological deterioration and activate a critical care response to the bedside to assess and intervene [5, 6]. Rapid response systems operate with an afferent arm, an early warning score or trigger mechanism and an efferent arm, a rapid response team. In the context of this review, rapid response teams are defined as critical care physician-led teams designed to provide rapid critical care for in-patients on general hospital wards. In recent years, rapid response systems have been adopted globally with multiple nations mandating their use in major hospitals [7–10].

Current evidence is mixed as to the effectiveness of rapid response systems at the reduction of patient mortality, with most evidence suggesting some effect at reducing cardiac arrest rates [4, 11–13]. Several single-center studies as well as a meta-analysis have found improved outcomes with rapid response system implementation [12, 14–16]. However, another meta-analysis and the only multicenter randomized control trial to date have not found strong evidence to support the effectiveness of rapid response systems [2, 3]. In many of these studies, the quality of the rapid response system as a systematic intervention itself has not been evaluated [17–20]. Previous reviews of rapid response systems have treated rapid response systems as if they were of equal quality and had comparable operating procedures [2, 17, 19, 21]. Few studies have reported on the response times of their efferent arms and how this may impact patient outcomes [13, 22, 23].

The timely identification and response to critical deterioration in patients is key to the effectiveness of a rapid response system at decreasing patient mortality, ICU admissions, and cardiac arrest rates [24]. Some studies have suggested that a delay between identification of deterioration and the rapid response team arriving is associated with a higher mortality [25, 26]. In addition, there currently exists no standardized guideline as to what constitutes a delayed activation of the rapid response system.

## Objectives

The primary objective of this systematic review is to identify and critically assess the existing literature assessing the effect of delayed activation of rapid response teams on hospital mortality among in-hospital patients. The secondary objective is to assess the effect of delayed activation of rapid response teams on cardiac arrests and ICU transfers. This will be conducted by examining the association between increased response times and mortality, ICU transfers, or cardiac arrest.

The secondary objective is to evaluate these studies for what they define as a delayed activation of a rapid response team, how rapid response teams are triggered, and if identifiable, where potential delays may occur in the activation process. The review will be reported according to the Preferred Report Items for Systematic Reviews and Meta-Analyses (PRISMA) guidelines [27].

## Methods

This systematic review protocol has been designed with the PRISMA-P guidelines for reporting systematic reviews in mind [27]. A checklist of PRISMA-P criteria met is included in Additional file 1. This protocol has been registered with the PROSPERO International Prospective Register of Systematic Reviews (PROSPERO CRD42017071842).

### Eligibility criteria

Included studies examined populations of hospitalized adult (≥ 18 years) patients that experienced a rapid response or medical emergency team call.

Studies assessing the effect of delayed activation, or response teams of rapid response teams or medical emergency teams will be considered for inclusion. These studies will be included if there are clear outlined criteria for what calling criteria would be for the activation of these teams, without any limitation on the afferent or triggering system. Studies must give reference to what constitutes a delayed or early call, or examine the relationship between response time and patient outcomes for inclusion. Studies must include a control group. Outcomes of interest are defined as of the following critical events: patient mortality, cardiac arrest, and ICU admission. These outcomes were selected as they are the outcomes for which rapid response teams seek to prevent and have been assessed against in previous studies [3, 18, 28]. There is no minimum number or percentage of patients that experience these outcomes needed for inclusion in this review. No exclusions will be placed on country; studies must be published in English.

Studies will be excluded if they meet any of the following criteria: do not report on patient outcomes following the arrival of a rapid response team, do not describe the criteria or methods for the activation of a rapid response team, do not report on quantitative data regarding the delayed activation (i.e., measures of association) or the length of delay, and/or are editorials or commentaries.

### Search strategy

The search strategy aims to find both published literature and any potential gray literature between January 1, 1990, and the time of the start of the review process. A three-step strategy will be utilized in this review. Initially, a limited search of the MEDLINE database will be undertaken to determine keywords of interest that may be used in the title, abstract, and indexing of relevant literature. A draft of the search strategy for MEDLINE can be found in Additional file 2. Following this, a second search using keywords identified previously will be undertaken across MEDLINE, EMBASE, Cochrane, and CINAHL. Additionally, full reference lists of included studies will be screened. A PRISMA flowchart will illustrate the study selection process and reasons for exclusion. Studies will be assessed for eligibility by one reviewer and checked by a second.

### Data abstraction

Data abstraction will be conducted independently by two reviewers (MX, KD). Data extracted will be entered into a spreadsheet. The following data items will be abstracted when available: (i) study identification items (first author, year of publication), (ii) study design characteristics (intervention, calling criteria for rapid response team, sample size, control group, defined time for delayed activation, duration of data collection), (iii) target population, (iv) setting (nationality, healthcare environment, maturity of response team), and (v) clinical outcomes (cardiac arrest, ICU admission, mortality).

### Risk of bias/quality assessment

In order to assess the quality of research, two independent reviewers (MX, KD) will assess the risk of bias using the Cochrane Collaboration’s tool for assessing risk of bias in RCTs, as well as the ROBINS-I assessment tool for non-RCT studies [29, 30]. Each study will be assessed for procedures specified in their respective appropriate tool. Studies will be rated as showing a “low,” “moderate,” or “high” risk of bias according to criteria specified in each tool.

### Data synthesis

Given the anticipated paucity of literature, published or otherwise on this topic, this systematic review is intended to be exploratory, inclusive, and descriptive in nature. The primary objective of this review is to identify and appraise literature regarding the delayed activation of rapid response systems. However, a meta-analysis may be considered dependent on the body of literature identified.

## Discussion

This systematic review will add to previous research on rapid response systems by synthesizing, summarizing, and discussing the existing literature on the effect that delayed activation of the rapid response team has on patient outcomes. To the authors’ knowledge, this is the first systematic review to specifically examine the impact that delayed activation has. Prior systematic reviews have evaluated rapid response systems or rapid response teams as interventions, but none have evaluated the effect of quality of these systems as an intervention and how degradation of their effectiveness impacts patient outcomes. The proposed review will provide a valuable overview and synthesis of a potential area for improvement and discussion regarding rapid response systems and their use.

The proposed review will go beyond summarizing the existing evidence, by also looking at factors listed contributing to delays in activation. In this way, areas needing further study can be identified and potential poor practices in the deployment of these systems can be highlighted.

Rapid response systems possess high face validity for being an effective systematic intervention for the early detection and management of critical deterioration in patients; however, the literature has provided mixed evidence for this effect. Given that these systems rely on rapid response, it is surprising that there is little literature regarding how to best implement and use these systems, especially with respect to response times. With the rapid adoption of these systems, it is crucial to determine how increased response times may degrade the effectiveness of rapid response systems at improving patient outcomes. The proposed systematic review is urgently needed and will substantially add to the current evidence, helping to shape and guide future practice regarding rapid response systems.

## Additional file

Additional file 1: PRISMA-P checklist. (DOCX 31 kb)

Additional file 2: Draft search strategy. (DOC 24 kb)
