# Supplementary material for: Evaluating the effect of delayed activation of rapid response teams on patient outcomes: a systematic review protocol
Source: Syst Rev. 2018 Mar 9;7:42. doi: 10.1186/s13643-018-0705-x (PMC5845146; doi:10.1186/s13643-018-0705-x)
Supplement: Supplementary file 2 — Draft search strategy. (DOC 24 kb) [file 13643_2018_705_MOESM2_ESM.doc]

Database: OVID Medline Epub Ahead of Print, In-Process & Other Non-Indexed Citations, Ovid MEDLINE(R) Daily and Ovid MEDLINE(R) 1946 to Present

Search Strategy:

--------------------------------------------------------------------------------

1 Time Factors/ (1123557)

2 Time-to-Treatment/ (3064)

3 (earl* or delay*).mp. [mp=title, abstract, original title, name of substance word, subject heading word, keyword heading word, protocol supplementary concept word, rare disease supplementary concept word, unique identifier, synonyms] (1975376)

4 1 or 2 or 3 (2920012)

5 rapid response team*.mp. [mp=title, abstract, original title, name of substance word, subject heading word, keyword heading word, protocol supplementary concept word, rare disease supplementary concept word, unique identifier, synonyms] (956)

6 rapid response system*.mp. [mp=title, abstract, original title, name of substance word, subject heading word, keyword heading word, protocol supplementary concept word, rare disease supplementary concept word, unique identifier, synonyms] (322)

7 medical emergency team*.mp. [mp=title, abstract, original title, name of substance word, subject heading word, keyword heading word, protocol supplementary concept word, rare disease supplementary concept word, unique identifier, synonyms] (510)

8 critical care outreach team*.mp. [mp=title, abstract, original title, name of substance word, subject heading word, keyword heading word, protocol supplementary concept word, rare disease supplementary concept word, unique identifier, synonyms] (32)

9 Hospital Rapid Response Team.mp. or Hospital Rapid Response Team/ (594)

10 5 or 6 or 7 or 8 or 9 (1451)

11 4 and 10 (507)

***************************
